# Wild-type and central DNA flap defective HIV-1 lentiviral vector genomes: intracellular visualization at ultrastructural resolution levels

**DOI:** 10.1186/1742-4690-3-38

**Published:** 2006-06-26

**Authors:** Nathalie J Arhel, Sylvie Souquere-Besse, Pierre Charneau

**Affiliations:** 1 Groupe de Virologie Moléculaire et Vectorologie, Institut Pasteur, 25–28 rue du Dr. Roux, 75724 Paris, France; 2 Institut André Lwoff, CNRS-FRE2937, 7 rue Guy Moquet-BP8, 94800 Villejuif, France

## Abstract

HIV-1 and other lentiviruses have the unique ability among retroviruses to efficiently replicate in non-dividing cells as a result of the active nuclear import of their DNA genome across an interphasic nuclear membrane. Previous work has shown that a three-stranded DNA structure synthesized during HIV-1 reverse transcription, called the central DNA flap, acts as a cis-determinant of HIV-1 genome nuclear import. Concordantly, DNA Flap re-insertion in lentiviral-derived gene therapy vectors stimulates gene transfer efficiencies and complements the level of nuclear import to wild-type levels quantitatively indistinguishable from wild-type virus in all cell types and tissues examined so far. In order to define the precise nature of the replicative defect of DNA flap mutant viruses, we carried out *in situ *DNA hybridization experiments with electron microscopy to determine the subcellular localization of DNA flap mutant and wild-type HIV-1 genomes. We found that Flap defective DNA genomes accumulate at the cytoplasmic face of the nuclear membrane with no overlap across the nuclear membrane, whereas wild-type genomes localize throughout the nuclear compartment. These data provide an unequivocal confirmation of the role of the DNA flap in HIV-1 nuclear import and further establish that the DNA flap controls a step that immediately precedes translocation through the nuclear pore. Further, the widespread distribution of wild-type genomes within the open chromatin confirms the recent genome-wide mapping of HIV-1 cDNA integration sites and points to an as-yet poorly understood step of intranuclear transport of HIV-1 pre-integration complexes.

## Findings

HIV-1 and other lentiviruses have evolved a more complex reverse transcription strategy than oncoviruses whereby the presence of two additional cis-acting sequences within the lentiviral genome, the central polypurine tract (cPPT) and the central termination sequence (CTS), leads to the formation of a three-stranded DNA structure called the central DNA Flap [1-3]. Mutations within the cPPT lead to a linear genome lacking the central DNA Flap and severely impair viral replication [2,4]. While wild-type viral DNA is almost entirely imported into the nucleus where it either integrates or circularizes, DNA Flap defective viral DNA accumulates as unintegrated linear DNA as a consequence of a lack of access to the nuclear compartment, indicating a defect in nuclear import [4].

Consistently with the cis-acting role of the central DNA Flap in HIV-1 genome nuclear import, its reinsertion in HIV-1 derived gene transfer vectors can complement the level of nuclear import from a strong defect to wild-type nuclear import levels, quantitatively indistinguishable from wild-type virus [4]. As a result, DNA Flap containing lentiviral vectors closely mimic the early steps of wild-type virus infection. Reinsertion of the DNA Flap in HIV-1 vectors stimulated gene transfer efficiencies both *in vivo *and *ex vivo *in all tissue- and cell-types examined [5-14], thus making the DNA Flap an essential and widely-used component of lentiviral gene therapy vectors.

In an effort to elucidate the mechanistic implication of the central DNA Flap in HIV-1 nuclear import, we sought to precisely characterize the nature of the nuclear import defect of DNA Flap mutant viruses. We previously found, using subcellular fractionation experiments together with localization of viral DNA by fluorescence *in situ *hybridization (FISH) that central DNA Flap defective molecules accumulate at close proximity to the nuclear compartment indicating a late defect in nuclear import following a normal and rapid routing process of viral complexes from the plasma membrane to the nuclear membrane [4]. However, the precise nature of the nuclear import replicative defect, such as translocation through the nuclear pore or any step that immediately precedes or follows translocation, was not known.

We therefore sought to define with ultrastructural resolution the subcellular compartment of accumulation of Flap+ versus Flap- HIV-1 genomes. We carried out *in situ *DNA hybridization with electron microscopy on MT4 cells transduced with HIV-1 derived vectors, including or not the central DNA Flap. MT4 cells are HTLV-1 transformed human CD4+ T lymphocytes, maintained in RPMI 1640 medium supplemented with 10% FCS. MT4 cells are highly permissive to transduction, while vectors benefit from higher titers compared to viruses, thus optimizing the detection of vector DNA within cell sections. In addition, the use of HIV-1 replication defective vectors enabled us to limit our observations to one-round transduction events. The HIV-1 vector HR, derived from HR'CMVLacZ [15], does not contain the cis-acting sequences required for formation of the central DNA Flap. The HIV-1 vector TRIP is identical to HR but with the DNA Flap sequences reinserted within the vector sequence. HIV-1 vectors were produced as previously described [15]. Carry-over DNA in the vector supernatants was eliminated by treating the vector stocks with DNase I (1 μg/ml in the presence of 1 μM MgCl_2_) for 15 min at 37°C.

MT4 lymphocytes were transduced with the TRIP Flap+ or HR Flap- vector, with a multiplicity of infection of 100, as determined from the number of copies of provirus per cell by quantitative PCR (a high MOI is required for viral DNA detection by *in situ *DNA hybridization with electron microscopy). At 48 hr post-transduction, a time point when most incoming complexes, in the context of a highly asynchronous infection, have completed the entire early steps phase and have reached their final state [4], transduced and non-transduced cells were fixed in 4% formaldehyde (Merck) in 0.1 M Sörensen's phosphate buffer, pH 7.3, at 4°C for 1 hr. These were then dehydrated in methanol and embedded at low temperature in Lowicryl K4M (Polysciences Europe, Germany). Polymerization was carried out under long wavelengh UV light at -30°C. Ultrathin sections were collected onto carbon-Formvar-coated gold grids (200 mesh). *In situ *DNA hybridization was carried out as previously described [16] using a biotinylated double-stranded vector specific DNA probe [4] (please refer to Additional file 1 for a detailed protocol for HIV-1 DNA genome detection by *in situ *DNA hybridization and electron microscopy). Prior to hybridization the grids underwent a series of successive enzymatic and denaturation treatments (Table 1): bacterial protease type VI (Sigma, St Louis, MO/USA) to render the DNA accessible to the probe, RNase A (BDH Biochemical Ltd, UK) to eliminate RNA sequences including those that are homologous to the probe, NaOH treatment to denature the DNA present in the sections, and heat treatment to denature the double-stranded DNA probe. Vector DNA-probe hybrids were detected within 90 nm thick sections by direct immunogold labeling using anti-biotin conjugated 10 nm colloidal gold particles (British Biocell International) diluted 1/25 in PBS, for 30 min at room temperature. Grids were stained with uranyl acetate prior to observation. The specificity of the hybridization signals was confirmed by negative results following additional DNase I treatment of sections prior to hybridization (1 mg/ml, Worthington Biochem. Corp.) at 37°C for 1 hr (data not shown), and *in situ *DNA hybridization of non-transduced cells (Figure 3A). Samples were observed with a Philips 400 electron microscope at 80 kV.

**Table 1:** Sequential experimental steps for *in situ *hybridization

*Step*	*Material*	*Concentration*	*Buffer*	*Duration*	*Temperature*
Fixation	Paraformaldehyde	4%	Sörensen	1 hr	4°C
Lowicryl embedding				5 days	-30°C
Sectioning (gold grids)					
Enzymatic digestion	Protease^a^	0.2 mg/ml	Distilled water	15 min	37°C
	RNase A^b^	1 mg/ml	Tris HCl, 10 mM, pH 7.3	1 hr	37°C
Denaturation of grid target DNA	NaOH	0.5 N	Distilled water	4 min	RT^d^
Denaturation of hybridization solution				4 min	95°C
Hybridization				o/n^c^	37°C
Detection of hybrids	Anti-biotin 10 nm gold conjugate	1:25	PBS	30 min	RT^d^

^a ^The aim of this step is to eliminate proteins within the section which could otherwise interfere with binding of the probe to the target DNA. ^b ^The aim of this step is to eliminate all RNA molecules including viral RNA to prevent their concomittant detection with viral DNA. ^c ^Overnight. ^d ^Room temperature

The images that we show were taken from 4 independent cell population infections and inclusions, and 25 hybridization experiments. In every experiment, each electron transmission grid contained 5 sample slices, each of which contained 10–20 cell sections. Therefore, approximately 1,250 to 2,500 cells were observed for the purpose of this study. In the context of HIV infection, detection by *in situ *DNA hybridization and immuno-gold staining is a rare event, even when using a permissive target cell such as MT4 and high multiplicity of infection. As a result, about 5–10% of cells observed actually contained DNA hybridization signal. Importantly, all cell areas containing DNA hybridization signal were systematically photographed (about 150–200 photos). Observations were carried out in single-blind conditions, and images shown are highly representative of all data obtained.

**Figure 1:**
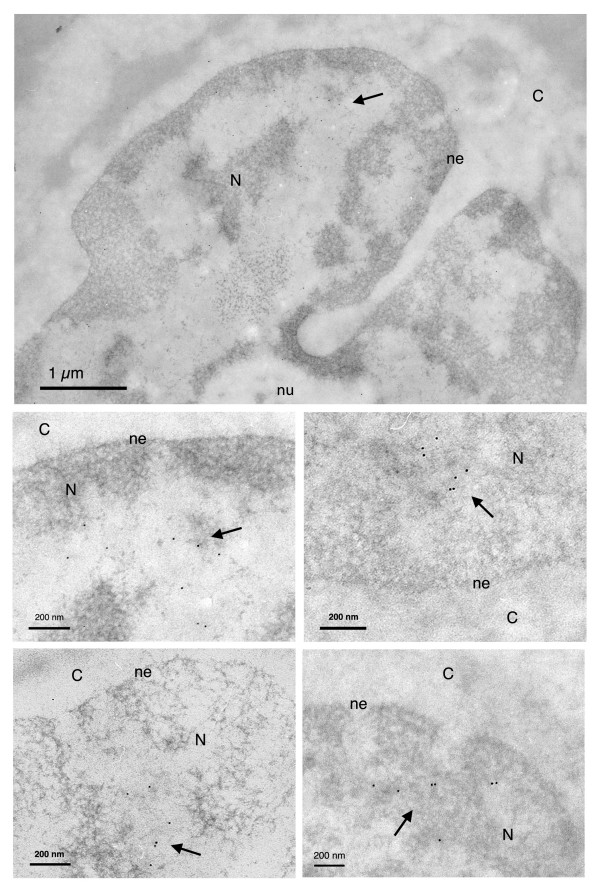
**Ultrastructural subcellular localization of HIV-1 derived vector genomes including the central DNA Flap (Flap +)**. Electron micrographs showing MT4 cells 48 hr following transduction with the TRIP Flap+ vector. Vector DNA genomes including the DNA Flap are found predominantly within the nucleus. N = nucleus; ne = nuclear envelope; nu = nucleolus; C = cytoplasm. Images show one low and four high magnification micrographs. The first high magnification micrograph is an enlargement from the low magnification image. The other three are taken from other independent experiments. All are highly representative of the data obtained. Arrows point to clusters of immunogold labeled vector DNA.

We found that wild-type vector DNA including the DNA Flap accumulates predominantly within the nucleus of transduced cells 48 hr post-transduction, and more specifically within open regions of the chromatin (Figure 1). The detection of vector DNA hybridization signals does not discriminate between integrated proviruses and DNA circles, therefore intranuclear signals do not all necessarily correspond to actively transcribed genomes. At 48 hr post-transduction, previous DNA profile analyses showed that ~55% of nuclear localized genomes are integrated proviruses, the rest being one-long terminal repeat (LTR) DNA circles and few 2-LTR circles [4].

**Figure 2:**
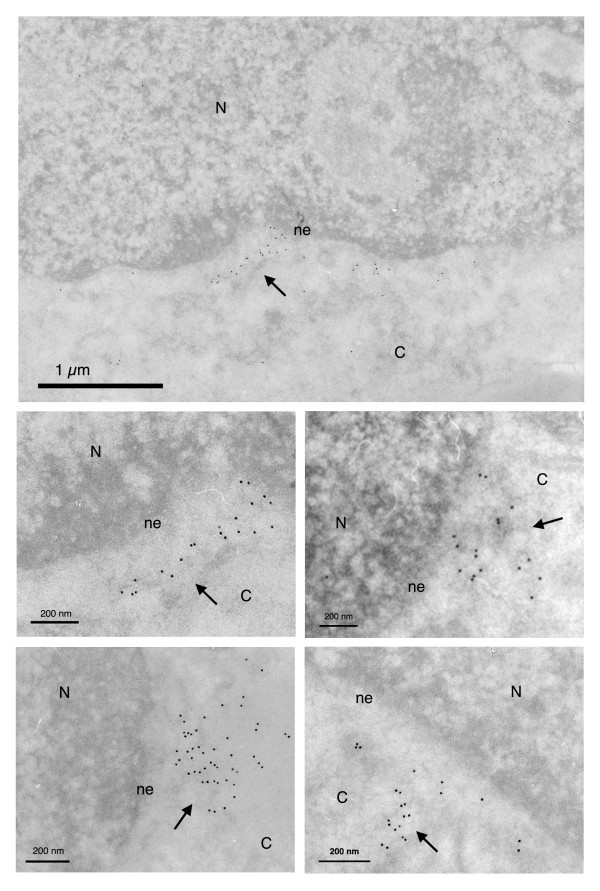
**Ultrastructural subcellular localization of HIV-1 derived vector genomes without the central DNA Flap (Flap -)**. MT4 cells 48 hr post-transduction with the HR Flap- vector. DNA Flap defective vector genomes localize on the cytoplasmic side of the nuclear membrane. N = nucleus; ne = nuclear envelope; C = cytoplasm. Images show one low and four high magnification micrographs. The first high magnification micrograph is an enlargement from the low magnification image. The other three are taken from other independent experiments. All are highly representative of the data obtained. Arrows point to clusters of immunogold labeled vector DNA.

**Figure 3:**
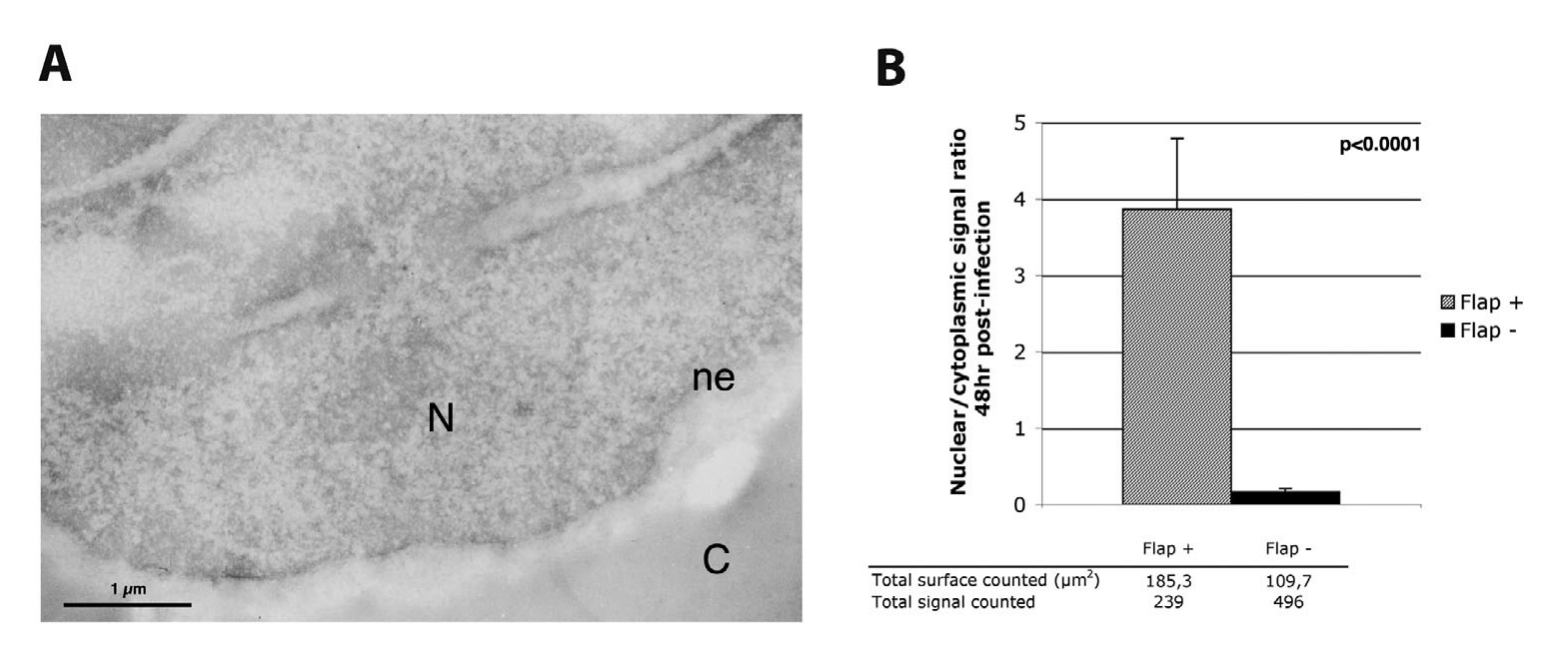
**Quantification of intracellular vector genome detection**. (A) Electron micrograph of control non-transduced cells showing minimal background signal. (B) DNA hybridization signals from 4 independent cell population infections were counted and represented as total nuclear over cytoplasmic signal ratio. All cell areas containing DNA hybridization signal were systematically photographed (about 150–200 photos) and signal was carefully quantified, each time with equal surface of nuclear and cytoplasmic compartments. The *p *value (Mann-Whitney test) shows the results are highly statistically relevant.

DNA Flap defective viral genomes, on the other hand, accumulate predominantly on the cytoplasmic face of the nuclear membrane (Figure 2), confirming a nuclear import defect of Flap defective pre-integration complexes that does not implicate the routing process from the plasma membrane to the nucleus. As previously shown, there is no degradation of this cytoplasmic linear unintegrated viral DNA between 12 and 48 hr post-transduction [4]. Importantly here, DNA hybridization signals remain on the cytoplasmic side of the nuclear membrane, with no overlap across the nuclear membrane, revealing that Flap defective DNA molecules have not initiated translocation through the nuclear pore. Non-transduced control samples exhibited negligible background signal (Figure 3A). Precise quantification of DNA hybridization signals 48 hr post-transduction on randomly selected micrographs (Figure 3B) revealed a strong accumulation of nuclear versus cytoplasmic vector DNA in the case of DNA Flap+ vector with an average nuclear/cytoplasmic ratio of 3.9 ± 0.9, which means that 77.3 ± 3.6% of total vector DNA was detected within the nuclear compartment. Conversely, there was a strong accumulation of cytoplasmic versus nuclear vector DNA in the case of DNA Flap- vector with an average nuclear/cytoplasmic ratio of 0.2 ± 0.04, which corresponds to 86.4 ± 2.8% of total vector DNA being detected in the cytoplasm. Results are highly statistically significant (p < 0.0001, Mann-Whitney test) and consistent with published intracellular vector DNA profiles assessed by quantitative Southern blotting [4].

This is, to our knowledge, the first report of the intracellular visualization of HIV-1 DNA genomes by *in situ *DNA hybridization with electron microscopy. The data we obtained reveal that lack of the central DNA Flap results in perinuclear accumulation of viral genomes that do not overlap across the nuclear membrane, indicating a defect preceding translocation of pre-integration complexes through the nuclear pore. Of note and as we previously reported [4], absence of the central DNA Flap does not entirely preclude HIV-1 genome nuclear import. The ~10–20% of Flap- genomes that are imported into the nucleus points to a Flap-independent nuclear import mechanism that likely accounts for transduction levels obtained with Flap- vectors, a current area of investigation in our laboratory.

The search for the viral determinants responsible for the active nuclear import of the HIV-1 DNA genome has constituted an active but controversial field of investigation. Based on the search of a sequence that obeys the consensus for nuclear localization, three HIV-1 proteins, MA, Vpr and IN, have been proposed to contribute in a redundant manner to the karyophilic properties of the HIV-1 pre-integration complex [[17-19], among others]. However, the actual participation of these proteins in HIV-1 genome nuclear import is a matter of strong debate [[20-23], among others]. The implication of the central DNA Flap in HIV-1 nuclear import has also been questioned in two reports [24,25] that suggested the central DNA Flap, while important in the context of HIV-1 derived vectors, not to be essential for HIV-1 replication. However, detailed analyses of virus infectivity revealed that all cPPT mutant viruses exhibit reduced infectivity and defective nuclear import irrespective of the viral genetic background or target cells (manuscript submitted). Here, the ultrastrucutral localization of Flap defective molecules confirms that the central DNA Flap is a cis-acting DNA motif that is implicated in HIV-1 nuclear import. The inhibition of nuclear import in the absence of this motif, although not absolute, points to a mechanistic implication of the DNA Flap in a step immediately prior to translocation of the viral genome through the nuclear pore. Other viral factors, and conceivably many cellular factors, are also expected to contribute to the active nuclear import of HIV-1.

In the case of Flap+ vector genomes, hybridization signals were detected predominantly within the open regions of the chromatin, confirming previous work showing preferential integration of HIV-1 within actively transcribed genes [26-28]. Moreover, signals are visualized throughout the nuclear compartment, without particular preference for areas close to the nuclear membrane, indicating probable intranuclear transport events of HIV-1 DNA genomes after translocation through the nuclear membrane and until the integration sites are reached. This concurs with the recent HIV-1 integration site mapping showing integration throughout the genome [26]. The nature and mechanisms of this intranuclear transport remain entirely to be characterized.

## Competing interests

The author(s) declare that they have no competing interests.

## Authors' contributions

NJA prepared the infected samples, participated in the design of the study, performed the statistical analysis, and drafted the manuscript. SSB carried out all hybridizations and electron microscopy observations in a single blind fashion, and contributed to the statistical analysis. PC conceived and coordinated the study and drafted the manuscript. All authors read and approved the final manuscript.

## Supplementary Material

Additional File 1 Detailed protocol for HIV-1 DNA genome detection by *in situ *DNA hybridization and electron microscopy. Click here for file
