# Supplementary material for: Wild-type and central DNA flap defective HIV-1 lentiviral vector genomes: intracellular visualization at ultrastructural resolution levels
Source: Retrovirology. 2006 Jun 26;3:38. doi: 10.1186/1742-4690-3-38 (PMC1538615; doi:10.1186/1742-4690-3-38)
Supplement: Additional File 1 — Detailed protocol for HIV-1 DNA genome detection by in situ DNA hybridization and electron microscopy. [file 1742-4690-3-38-S1.doc]

**Detailed protocol for HIV-1 DNA genome detection by *in situ* DNA hybridization and electron microscopy**

*HIV-1 vector transduction and sample fixation*

MT4 lymphocytes were transduced with the TRIP Flap+ or HR Flap- vector, with a multiplicity of infection (MOI) of 100. Transduced and non-transduced cells were fixed at 48 hr post-transduction in 4% formaldehyde (Merck) in 0.1 M Sörensen’s phosphate buffer, pH 7.3, at 4 °C for 1 hr at 4°C. After fixation, cells were centrifuged at low speed (800g). Cell pellets were then dehydrated in increasing concentrations of methanol and embedded at low temperature in Lowicryl K4M (Polysciences Europe, Germany) as previously described (Roth et al., 1981). Polymerization was carried out under long wavelengh UV light at -30°C for 5 days, and subsequently at room temperature for 1 day. Ultrathin sections were collected onto carbon-Formvar-coated gold grids (mesh 200).

*Pre-treatment of grids bearing Lowicryl sections*

Enzymatic digestions were carried out to improve the accessibility of the probe to the target sequence (Bacterial protease type VI (Sigma, St Louis, MO/USA)) and to eliminate binding of the probe to related RNA or DNA sequences (RNase A (BDH Biochemical Ltd, UK)). The compositions of the enzymatic solutions are given in **Table 1**. Each enzymatic reaction was performed at 37°C in a wet chamber by floating the gold grids bearing the Lowicryl sections onto 10 μl drops of enzymatic solution on a sheet of Parafilm. Grids were then rinsed in distilled water and air-dried.

Denaturation of cellular and viral DNA in the sections was carried out by NaOH treatment as indicated in **Table 1**. Grids were floated onto 10 μl drops of 0.5 N NaOH on a sheet of Parafilm, for 4 min, at room temperature. They were then rinsed in distilled water and air-dried. Grids were used for *in situ* hybridization within 10 min from the end of the denaturation step.

*In situ DNA hybridization*

*In situ* DNA hybridization was carried out using a biotinylated double-stranded vector specific DNA probe (Zennou et al., 2000). The 50 μl hybridization solution consisted of 50% deionized formamide, 10% dextran sulfate, 2 x SSC buffer, 400 μg/ml competitor dsDNA, and 10 μg/ml dsDNA probe. This solution was denatured by heat treatment (in boiling water for 4 min) immediately prior to hybridization. For the hybridization step, dried grids were floated onto microdrops (1 μl) of freshly denatured hybridization solution on a sheet of Parafilm at 37°C in a moist chamber for 90 min. Grids were then washed in phosphate buffered saline (PBS) and immediately incubated for 30 min at room temperature onto 5 μl drops of anti-biotin antibody conjugated with 10 nm colloidal gold particles (British Biocell International) diluted 1/25 in PBS. Grids were washed twice in PBS, once in distilled water and air-dried for 15 min at room temperature.

*Electron microscopy observation*

# Grids were stained for 10 min with 5% aqueous uranyl acetate prior to observation. Observations were carried out with a Philips 400 electron microscope at 80 kV, at 13,000 or 17,000 magnification.

## Table 1 - Sequential experimental steps for *in situ* hybridization


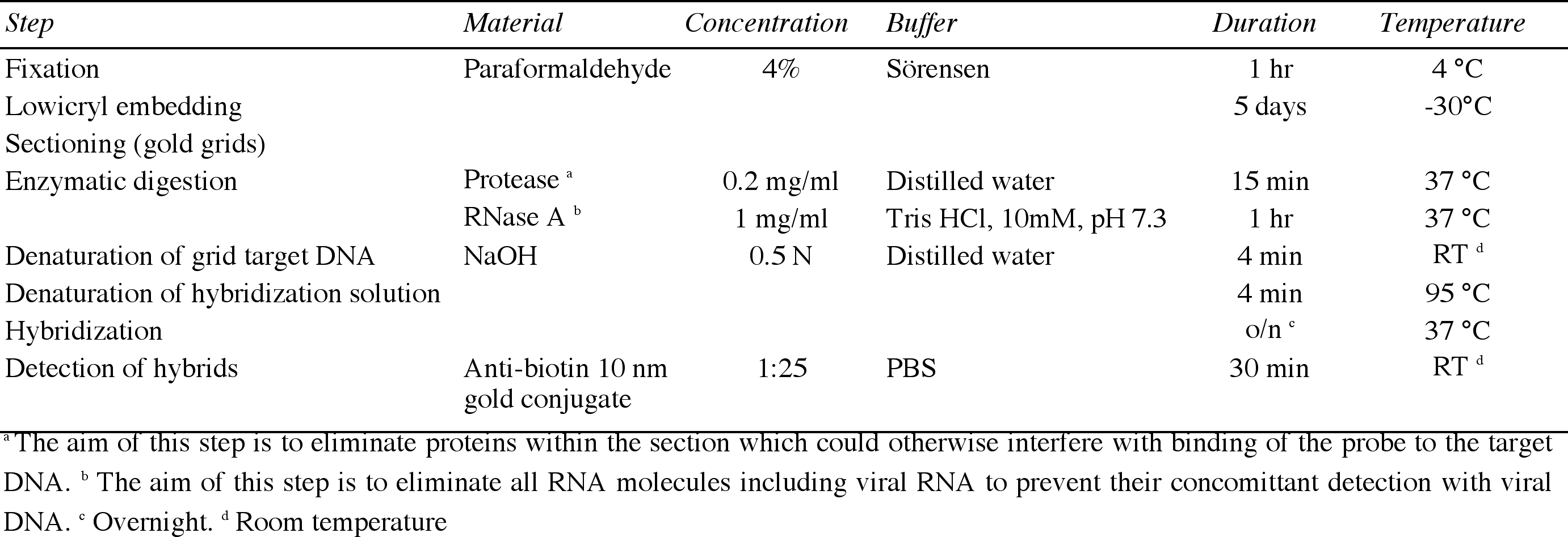


## References:

[Roth J, Bendayan M, Carlemalm E, Villiger W, Garavito M:](http://www.ncbi.nlm.nih.gov/entrez/query.fcgi?cmd=Retrieve&db=pubmed&dopt=Abstract&list_uids=6166664&query_hl=3&itool=pubmed_docsum) Enhancement of structural preservation and immunocytochemical staining in low temperature embedded pancreatic tissue. *J Histochem Cytochem.* 1981 29: 663-671.

Zennou V, Petit C, Guetard D, Nerhbass U, Montagnier L, Charneau P: HIV-1 genome nuclear import is mediated by a central DNA Flap. *Cell* 2000, 101: 173-185
